# Health literacy, multimorbidity and its effect on mental health in South African adults: a repeated cross-sectional nationally representative panel study

**DOI:** 10.3389/fpubh.2025.1622005

**Published:** 2025-08-20

**Authors:** A. Craig, K. Mabetha, O. Gafari, S. A. Norris

**Affiliations:** ^1^SAMRC/Wits Developmental Pathways for Health Research Unit, Faculty of Health Sciences, University of the Witwatersrand, Johannesburg, South Africa; ^2^DSI-NRF Centre of Excellence in Human Development, University of Witwatersrand, Johannesburg, South Africa; ^3^School of Health Sciences, University of Southampton, Southampton, United Kingdom; ^4^NIHR Southampton Biomedical Research Centre, University Hospital Southampton NHS Foundation Trust, Southampton, United Kingdom; ^5^School of Human Development and Health, University of Southampton, Southampton, United Kingdom

**Keywords:** depression, anxiety, ACE, mental health, multimorbidity, South Africa, national representative survey, health literacy

## Abstract

**Objective and methods:**

Health literacy is a key determinant of physical and mental health outcomes, particularly in low- and middle-income settings like South Africa, where multimorbidity is increasingly common. Limited health literacy may hinder effective management of multiple chronic conditions and worsen mental health. Using repeated cross-sectional nationally representative data, this study examined the relationship between health literacy and multimorbidity, with a specific focus on mental health among South African adults (18 years and older).

**Results:**

Most respondents had minimal depression risk, with 21.7% showing probable depression, lower than the 25.7 and 26.2% in Panels 1 (2021) and 2 (2022) respectively. In efforts to further corroborate the odds of having mental or physical health risk with higher levels of ACE exposure, our results confirmed the increased likelihood of depression, anxiety and multimorbidity with increased odds of early adversity, irrespective of differing socio-demographics. The results further revealed that socioeconomic status directly influenced depression, which was partially mediated via health literacy. Additionally, the association between socioeconomic status and multimorbidity was fully mediated by ACE exposure and depression.

**Conclusion:**

One in five South Africans experience depressive symptoms, with notable regional differences. Childhood adversity contributes to increased mental health risk and higher multimorbidity. Health literacy was found to influence the link between socioeconomic status and depression, suggesting that lower literacy increases vulnerability. These findings therefore emphasize the need for targeted interventions to address childhood adversity, improve health literacy, and enhance mental health resources across South Africa.

## Introduction

Health literacy is a significant driver of health outcomes which influences individuals’ ability to comprehend, access and utilize health information effectively. In South Africa, where disparities in access to quality healthcare persist (1), health literacy plays a pivotal role in shaping not only physical health outcomes (e.g., multimorbidity) but mental health, particularly in relation to conditions such as depression and anxiety. This relationship becomes even more complex when considering the impact of early adversity, which can predispose individuals to both mental health challenges (2–6) and difficulties in navigating healthcare systems in adulthood (7, 8). South Africa – a country characterized by a diverse population facing various socioeconomic challenges – highlights the need to investigate the intricate links between health literacy, multimorbidity, and mental well-being.

One tool used to assess health literacy is the European Health Literacy Questionnaire (HLS-EU-Q47), a comprehensive tool that captures individuals’ ability to access, understand, and apply health information across three domains: healthcare, disease prevention, and health promotion (9). Research has consistently shown that individuals with higher health literacy are better equipped to make informed health decisions, adhere to treatment regimens, and engage with healthcare professionals (10). Conversely, poor health literacy has been correlated with negative health outcomes, including a rise in hospitalisations and the prevalence of chronic disease and mental ill-health (11). Nationally, we have previously reported that the burden of mental health is alarmingly high in South African adults (12, 13), therefore understanding how health literacy influences mental health outcomes is crucial for developing effective interventions and preventative strategies.

In recent years, the interplay between health literacy, multimorbidity and mental health has gained attention, with research suggesting that individuals with lower health literacy are at a heightened risk for mental ill-health (14). Despite growing awareness of the importance of health literacy, there remains a gap in research specifically addressing its impact on mental health among South African adults. Prior studies have primarily focused on physical health, often neglecting the interplay between health literacy and mental health outcomes (13, 15, 16). This oversight is particularly concerning given the stigma surrounding mental health in South Africa (17), which can hinder individuals from seeking help and exacerbate their conditions.

This nationally representative study therefore aimed to explore the relationship between health literacy and multimorbidity, with a specific focus on mental health among South African adults aged 18 yrs. and older. Specifically, we sought to: 1. examine the associations between health literacy, multimorbidity, and mental health outcomes; and 2. assess the influence of socioeconomic status (SES), health literacy, and mental and physical health. By examining how health literacy influences both physical and mental health outcomes, we seek to provide insights that can inform public health strategies and interventions aimed at improving overall health and well-being in this diverse population. The use of nationally representative data enables a broader understanding of these associations across diverse demographic and socioeconomic groups. Through this research, this study aims to contribute to a better understanding of the role of health literacy in promoting mental health and to highlight the need for tailored health education initiatives that address the unique challenges faced by many South Africans.

## Methodology

### Study design

**Figure 1:**
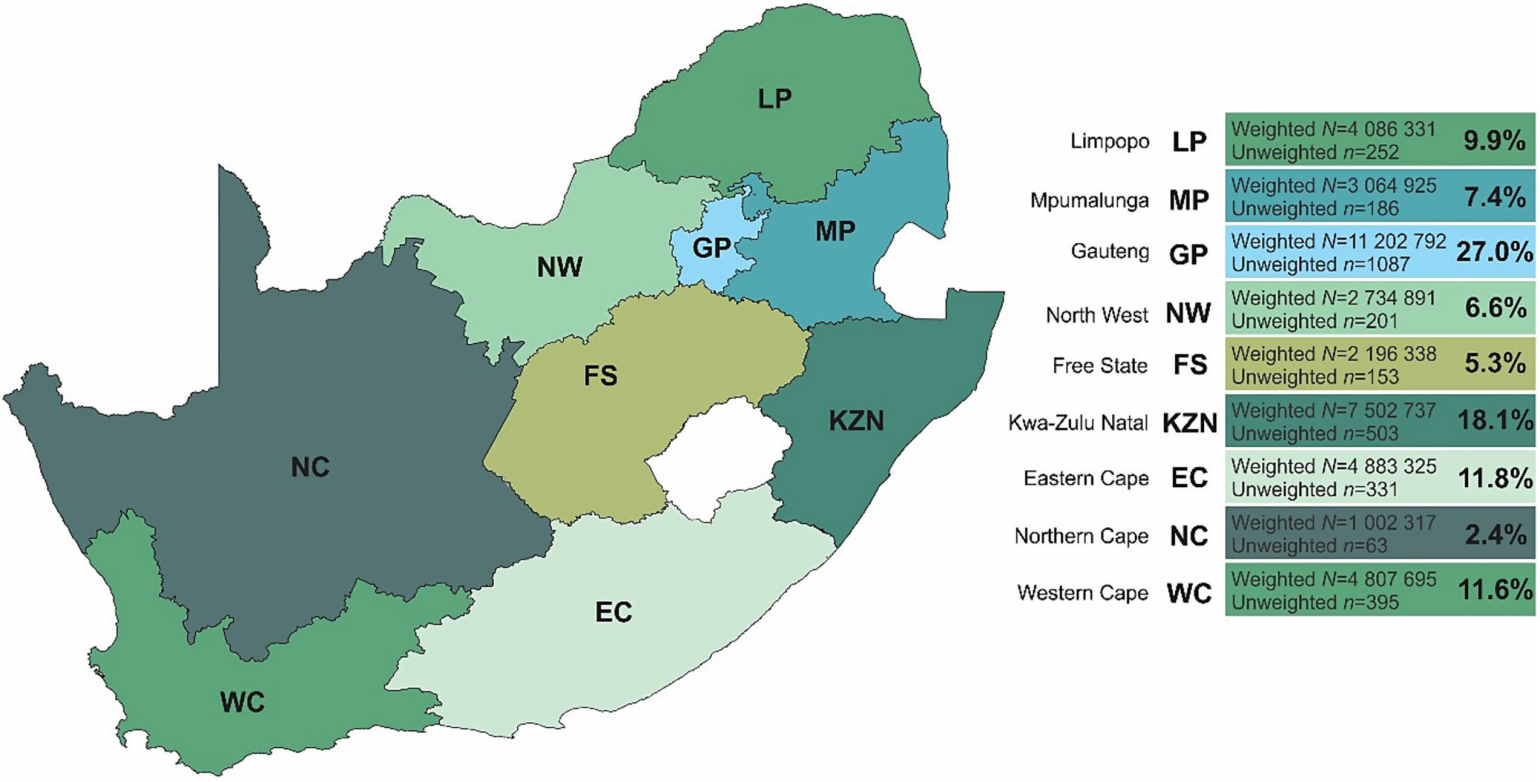
Population demographics outlining the nine provinces of South Africa.

This is a repeated cross-sectional study conducted in April 2024 [now referred to as Panel 3], that followed the first and second nationally representative panels conducted in September–October 2021 (Panel 1: *n* = 3,402) and May–June 2022 (Panel 2: *n* = 3,459) respectively (12, 13). In Panel 3, face-to-face interviews were conducted with 3,171 respondents in all provinces across South Africa (Figure 1).

Respondents provided written informed consent as well as the study obtained ethical approval from the Human Research Ethics Committee (Non-Medical) of the University of the Witwatersrand, South Africa (H21/06/36). Data collection followed the same collection procedures as previously outlined in Panel 1 (12) and Panel 2 (13).

### Survey questionnaires

All data were captured in real time using tablets and managed centrally through a secure database system. Respondent household demographic information (age, gender, marital status, employment, education level and household assets) was collected. In this study, a household asset score was subsequently computed in alignment with the Demographic and Health Surveys household questionnaire and used as an indicator of socioeconomic status (SES). This involved counting all key functioning household amenities (e.g., refrigerator, washing machine, television, computer, etc.). In this repeated cross-sectional study, tertiles of the household asset score were additionally calculated and used as a measure of economic differentiation (18–20). Information related to respondent provincial and community size (metropolitan, city/town, rural/village) were additionally recorded.

To assess the respondent’s level of mental health (depression or anxiety), the Patient Health Questionnaire (PHQ-9) (21) and the Generalized Anxiety Disorder (GAD-7) scale was utilized (22). These scales include either 9 (PHQ-9) or 7 (GAD-7) questions, with responses rated on a four-point Likert scale ranging from 0 (“Not at all”) to 3 (“Nearly every day”). Depression levels were classified into five categories: minimal, mild, moderate, moderately severe, and severe, based on PHQ-9 scores of 0–4, 5–9, 10–14, 15–19, and 20–27, respectively (21). Anxiety levels were classified into four categories: minimal, mild, moderate and severe, based on GAD-7 scores of 0–4, 5–9, 10–14 and15–21, respectively (22). Binary depression or anxiety was classified as a PHQ-9 or GAD-7 score of 10 or higher, respectively (21, 22). Additionally, to assess adverse childhood experiences (ACEs) of each respondent, a 12-item ACE questionnaire was used, which collected an individuals’ retrospective report of specific adversities encountered during the first 18 years of life (23). Exposure to ACEs was defined through 12 types of experiences grouped into three categories: emotional and/or physical abuse, sexual abuse, and household dysfunction (23). An overall ACE score was subsequently calculated based on the number of affirmative responses. The ACE score was further classified into three exposure groups, namely, 0 (no exposure), 1–3 (intermediate exposure), and 4–12 (high exposure) based on previous ACE scoring (24).

To evaluate the overall health of the respondent, individuals were asked a series of health-related questions regarding known chronic conditions (heart attack, stroke, high cholesterol, diabetes, overweight/obesity, HIV/AIDS, asthma/chronic obstructive pulmonary disease, joint/muscle problems (e.g., arthritis, gout), tuberculosis, cancer, liver disease, mental health issues (e.g., depression, anxiety, bipolar disorder), chronic kidney disease, and hypertension/high blood pressure). As multimorbidity is commonly defined as the presence of multiple, often chronic, health conditions that coexist, an overall multimorbidity score was calculated based on the total number of chronic conditions a respondent indicated, including mental health. The multimorbidity score was further categorized into three groups, namely, group 1 (0–1 conditions), group 2 (2 conditions), and group 3 (≥3 conditions).

Health literacy was measured using the HLS-EU-Q47, which includes three distinct groups of questions covering healthcare, disease prevention, and health promotion (9). The HLS-EU-Q47 uses a four-point Likert scale, ranging from 1 (“very difficult”) to 4 (“very easy”). Respondents who answered “I do not know” to any of the 47 questions were assigned a score of zero. The overall score was subsequently converted into a scale of 50 and further categorized into four levels, namely, 0–25 (inadequate), >25–33 (problematic), >33–42 (sufficient), and >42–50 (excellent). The HLS-EU-Q47 instrument has not been validated in a South African context, however, in our population, the Cronbach’s Alpha was calculated at 0.972 which is indicative of excellent internal consistency.

### Statistical analyses

For all statistical analyses, IBM® SPSS® version 29 (IBM Corporation, Armonk, New York), Stata® version 18.0 (StataCorp, College Station, TX, USA), and GraphPad Prism version 5.03 for Microsoft® Windows (GraphPad Software, San Diego, California, USA) were employed to analyze and visualize the data. Additionally, QGIS (Penn Libraries, Philadelphia, PA) was used to map and scale the geographical location of South Africa (Figure 1).

All statistics were weighted to reflect the most recent census of the South African population aged 18 years and older. The weighting matrix accounted for factors such as age, sex, population group, home language, and provincial distribution to equate for national representativity. In stratified analyses, post-stratified weights were not used as the primary objective was to assess associations within subgroups rather than to produce population level estimates. As such, results reflect weighted estimates within strata based on the survey sample. Proportions across sociodemographics (age, sex, marital status, education level, employment, SES, and urbanicity) and provinces were calculated using crosstabs, with significant differences identified by Chi-square tests and presented as percentages. Univariate and multivariable adjusted binary logistic regressions were performed to assess the odds of probable depression (PHQ-9 score ≥10), probable anxiety (GAD-7 score ≥10), and multimorbidity (chronic conditions excluding mental health) in adulthood, considering either ACEs or ACEs combined with several sociodemographics (age, sex, marital status, education level, employment, SES, and urbanicity) as confounders.

A generalized structural equation model (gSEM) was constructed *a priori* to examine the relationship between SES, mental health, and whether this relationship was mediated by health literacy and/or multimorbidity. Direct (unmediated), indirect (mediated), and total effects were computed and recorded, with the proportion of the total effect mediated also calculated. Pathways were modified and variables added or removed iteratively, with the Akaike Information Criterion (AIC) and Bayesian Information Criterion (BIC) of each model being compared. The final model was chosen based on its low information criterion values and high theoretical relevance. Direct, indirect, and total effects were estimated using non-linear combination methods.

To prevent multicollinearity in our statistical models, mental health was excluded from the multimorbidity score in cases where it correlated with mental health variables (i.e., any models that included depression, anxiety, or ACEs as dependent or independent variables).

We used the Social Determinants of Health as a theoretical framework, specifically the Fundamental Cause Theory (25), which was particularly relevant for our data analysis; this model aligned well with the construction of the *a priori* gSEM model, as it accounted for the impact of socio-economic factors (SES) on health outcomes (multimorbidity) while considering psychosocial (mental health) and cognitive-behavioral (health literacy) mediators.

## Results

### Repeated cross-sectional analysis

The full characteristics and associations of Panel 1 (12) and Panel 2 (13) have been described elsewhere. In this current sample, a total of 3,171 respondents (female: 50.4%; male: 49.6%) were included for repeated cross-sectional analysis (Table 1). Respondents were predominantly young adults aged 25–34 years (29.6%). The largest proportion of respondents were those who reported a marital status of single (62.9%), employed (47.2%), an education level of graduated high school or equivalent (53.1%), and resided in the Gauteng province [*n* = 1,087 (27.0%)] (Figure 1). When compared to Panels 1 (2021) and 2 (2022), respondent demographics were closely aligned to both prior panels.

**Table 1:** General descriptives of the South African survey respondents (*n* = 3,171).

		Unweighted	Weighted
Age categories			
18–24 years	%	18.6	15.1
25–34 years	%	29.6	27.8
35–44 years	%	26.2	22.7
45–54 years	%	14.6	14.6
55–64 years	%	6.9	12.0
65 + years	%	4.1	7.8
Education			
Uneducated/Partial primary	%	1.9	3.6
Primary school	%	2.5	3.8
Partial secondary	%	26.3	27.8
NSC/Short course	%	53.1	49.7
Tertiary	%	16.2	15.1
Socio-economic status			
Lower tertile	%	33.0	36.2
Middle tertile	%	39.6	38.8
Upper tertile	%	27.4	25.0
Employment status			
Unemployed	%	39.4	39.3
Employed	%	47.2	44.3
Student	%	7.5	6.1
Retired	%	5.9	10.4
Marital status			
Single	%	62.9	57.5
Married/Co-habit	%	30.6	33.0
Widowed/Divorced	%	6.5	9.5
Depression categories			
Minimal	%	54.5	56.0
Mild	%	23.0	22.4
Moderate	%	14.8	14.3
Moderately severe	%	5.8	5.6
Severe	%	2.0	1.8
Probable depression	%	22.6	21.7
Anxiety categories			
Minimal	%	60.6	62.0
Mild	%	24.7	24.3
Moderate	%	11.5	11.2
Severe	%	3.2	2.6
Probable anxiety	%	14.7	13.8
ACE score			
No exposure (*n* = 1,639)	%	51.7	51.1
Intermediate exposure (*n* = 983)	%	31.0	31.8
High exposure (*n* = 549)	%	17.3	17.0
ACE types			
Household dysfunction	%	40.3	40.1
Emotion/physical abuse	%	35.2	35.9
Sexual abuse	%	4.7	4.8
Urbanicity			
Metropolitan	%	53.3	41.9
City/Town	%	21.1	27.7
Rural/Village	%	25.6	30.5
Multimorbidity (chronic conditions incl. mental health)			
0–1 morbidity	%	86.7	83.8
2 morbidities	%	7.3	8.4
3 + morbidities	%	6.0	7.7
Sex			
Male	%	49.6	47.7
Female	%	50.4	52.3
Health literacy			
Inadequate	%	8.6	8.4
Problematic	%	22.3	21.9
Sufficient	%	52.2	53.0
Excellent	%	16.7	16.7

Probable depression was categorized into 5 groups based on scoring in the range 0–4 (minimal), 5–6 (mild), 10–14 (moderate), 15–19 (moderately severe), and 20–27 (severe). Probable anxiety was categorized into four groups based on scoring in the range 0–4 (minimal), 5–9 (mild), 10–14 (moderate), and 15–21 (severe). The ACE score was categorized into 3 exposure groups based on scoring 0 (no exposure); 1–3 (intermediate exposure) and 4–12 (high exposure). Multimorbidity score was categorized into 3 groups based on those respondents who reported null or one ailment (0–1 morbidity); those with comorbidity (2 morbidities); and those with multimorbidity (3 morbidities). *n*, number of participants; %: percentage; ACE, adverse childhood experience; NSC, national senior certificate.

### Mental health outcomes

**Figure 2:**
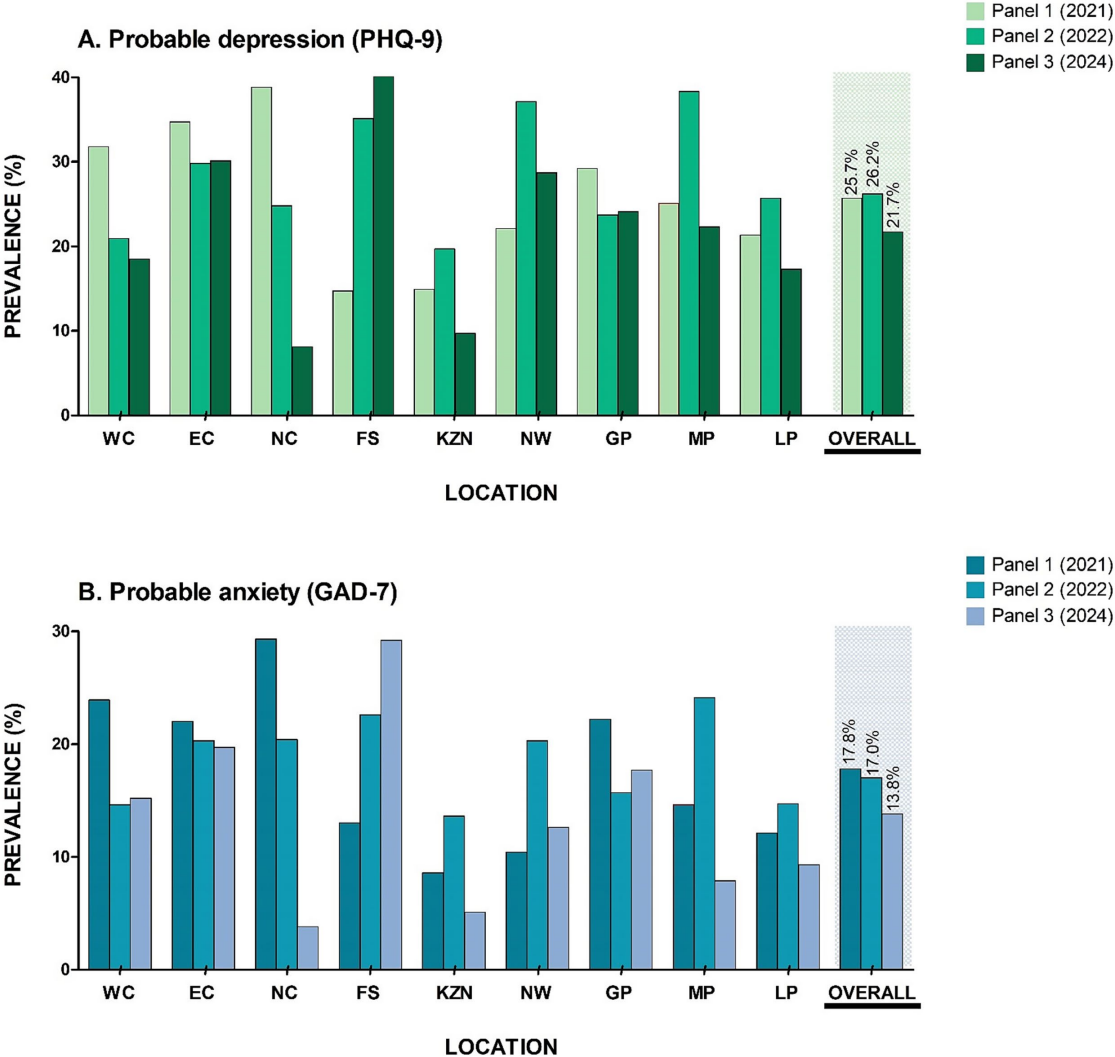
Prevalence of mental health risk across the South African provinces **(A)** probable depression and **(B)** probable anxiety. NC, Northern Cape; WC, Western Cape; NW, North West; GP, Gauteng province; LP, Limpopo province; MP, Mpumalanga province; FS, Free State; KZN, Kwa-Zulu Natal and EC, Eastern Cape.

Majority of the respondents scored as minimal risk (56.0%), while those scoring in more severe depressive categories were 22.4% (mild); 14.3% (moderate), 5.6% (moderately severe) and 1.8% (severe) respectively. We calculated an overall probable depression prevalence of 21.7% across South Africa (Table 1; Figure 2) which is lower than that reported in Panel 1 (25.7%) and Panel 2 (26.2%). Contrary to Panel 1 and Panel 2, Eastern Cape province reported the highest prevalence of probable depression (30.1%), and Free State province reported the highest probable anxiety (29.2%) (Figure 2; Supplementary Table S1). Childhood adversity was highest in Limpopo province with a mean score of 2.29 (SD: 2.26) (Supplementary Table S1). Additionally, comparable to Panel 2, the most common type of ACE reported was household dysfunction (40.1%) (Table 1).

### Socio-demographic correlates

Supplementary Table S2 presents the prevalence of mental health and multimorbidity among respondents stratified by socio-demographic determinants. In line with Panels 1 and 2, the prevalence of probable depression and anxiety was higher in women (≥14.0%); widowed, divorced, or separated (≥17.7%) and/or with only a basic level of education, i.e., primary school and/or partial secondary school (≥18.3%). Comparable to Panel 1, probable depression was highest in those who were unemployed (25.3%). Respondents in Panel 3 also showed a comparable higher prevalence of probable depression and high ACE exposure in those respondents with an SES score in the lowest tertile (≥19.2%). In contrast to Panels 1 and 2, probable depression was higher in middle aged adults (45–54 years: 25.4%), probable anxiety was highest in those aged 35–44 years (16.9%) and those in the highest ACE exposure groups were men (17.9%); widowed, divorced or separated (20.0%); in the age range of 25–34 years (18.7%), currently retired (20.0%). Multimorbidity of 3 or more morbidities across the socio-demographics was comparable to Panel 2 (65 + years: 23.4%; women: 8.6%; widowed, divorced or separated: 17.7%; retired: 21.1%).

### Associations of mental health risk, multimorbidity and socio-demographics

We repeated the univariate and multivariable adjusted binary logistic regressions (Supplementary Table S3) as seen in both Panels 1 and 2 to further corroborate the odds of having either probable depression or anxiety with higher levels of ACE exposure (model 1), or having ACE exposure, independent of socio-demographic characteristics (model 2). We confirmed the results determined in both panels showing that the likelihood of having probable depression (OR, 1.123 [95% CI 1.123; 1.124]) or probable anxiety (OR, 1.122 [95% CI 1.121; 1.122]) increases with each standard deviation increase in the ACE score (*p* < 0.001), independent of several socio-demographic determinants. We also reassessed the odds of having a higher number of morbidities with higher levels of ACE exposure (Supplementary Table S4) and confirmed that, respondents who reported having 0–1 morbidities were 80% less likely (OR, 0.796 [95% CI 0.796; 0.796]) to have experienced ACEs (*p* < 0.001) while, the likelihood of having 2 morbidities or 3 or more morbidities increased by 19% (OR, 1.193 [95% CI 1.193; 1.194]) or 21% (OR, 1.205 [95% CI 1.205; 1.206]) respectively, with each standard deviation increase in the ACE score (*p* < 0.001).

### Additional cross-sectional analysis

**Figure 3:**
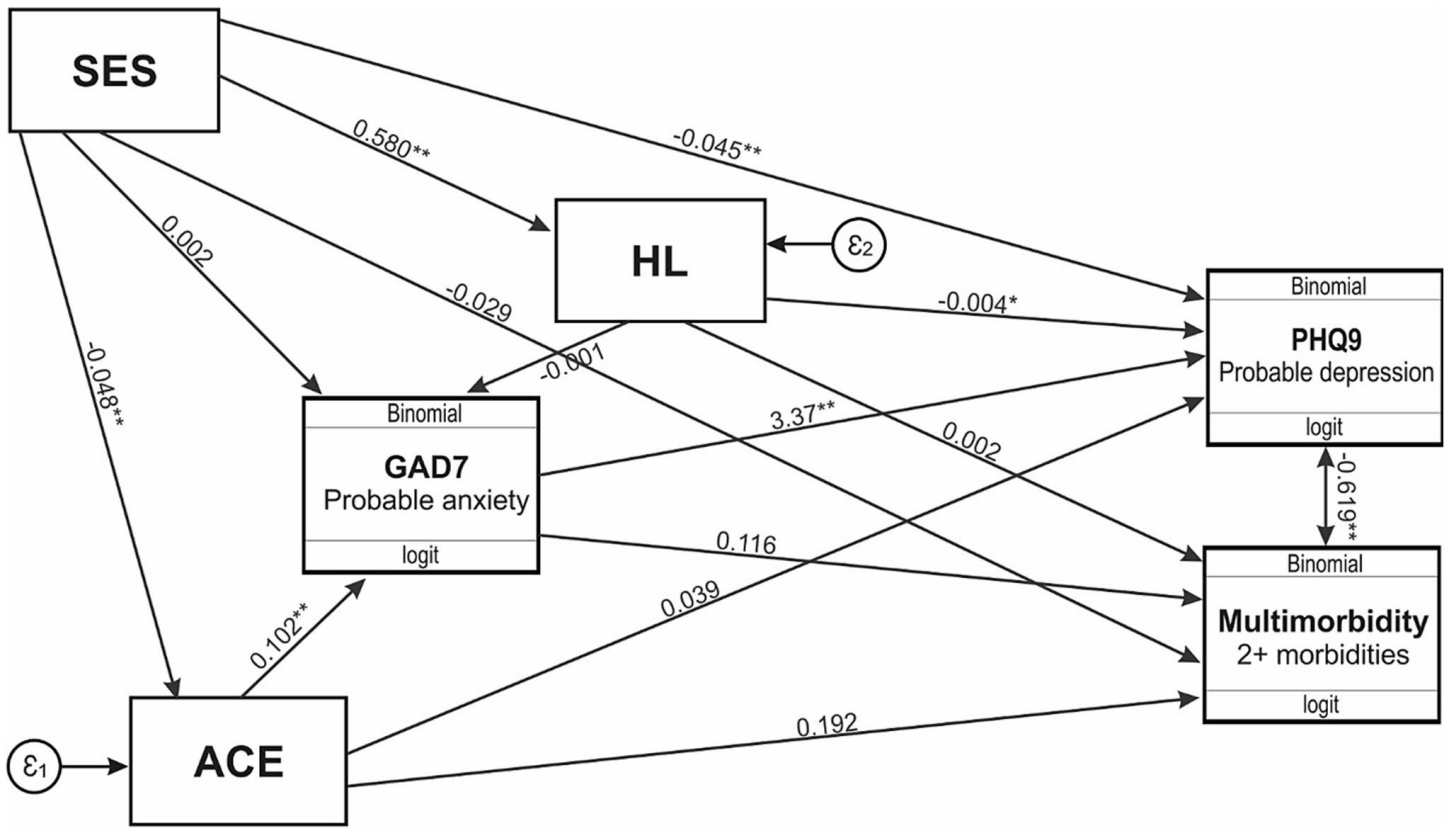
Generalized structural equation model for SES, health literacy, multimorbidity, and mental health. SES, socioeconomic status; HL, health literacy; PHQ9, Patient Health questionnaire 9; GAD7, Generalized Anxiety Disorder 7; ACE, adverse childhood experiences. * *p* < 0.05; ** *p* ≤ 0.00.

To assess the impact of SES, health literacy, multimorbidity, and mental health (Figure 3; Table 2), a gSEM was constructed *a priori*. In this model, SES was specified to influence both the outcomes (depression and multimorbidity) and the mediators (childhood adversity, anxiety, and health literacy), with childhood adversity and health literacy also modeled as influencing anxiety. The results revealed significant direct effects, specifically, SES on depression (*p* < 0.001), and ACE on multimorbidity (*p* < 0.001). The relationship between SES and depression was partially mediated by health literacy (4.2%) and ACE exposure (4.3%). In contrast, the impact of SES on multimorbidity was fully mediated by ACE (23.7%) and depression (54.7%). Furthermore, ACE was found to have a significant indirect and total effect on depression (*p* < 0.001), fully mediated by multimorbidity (75.3%) and anxiety (89.8%). Additionally, the effect of ACE on multimorbidity was partially mediated by depression (15.3%). These findings therefore highlight the complex interrelationships among these variables.

**Table 2:** Generalized structural equation model in a sample of respondents for socioeconomic status, health literacy, multimorbidity and mental health.

Exposure	Outcome	Direct effect		Indirect effect		Total effect		Proportion of total effect mediated
		Estimate (95% CI)	*p* value	Estimate (95% CI)	*p* value	Estimate (95% CI)	*p* value	
Effect of SES on depression via HL								
SES	PHQ9 < 10	Reference		Reference		Reference		-
	PHQ9 > 10 (probable)	−0.045 (−0.073; −0.018)	0.001	−0.002 (−0.005; −0.000)	**0.046**	−0.048 (−0.075; −0.020)	**0.001**	4.2%†
Effect of SES on depression via anxiety								
SES	PHQ9 < 10	Reference		Reference		Reference		-
	PHQ9 > 10 (probable)	−0.045 (−0.073; −0.018)	**0.001**	0.025 (−0.063; 0.113)	0.58	−0.020 (−0.113; 0.072)	0.67	-
Effect of SES on depression via ACE								
SES	PHQ9 < 10	Reference		Reference		Reference		-
	PHQ9 > 10 (probable)	−0.045 (−0.073; −0.018)	**0.001**	−0.002 (−0.004; 0.000)	**0.090**	−0.047 (−0.075; −0.020)	**0.001**	4.3%†
Effect of SES on depression via multimorbidity								
SES	PHQ9 < 10	Reference		Reference		Reference		-
	PHQ9 > 10 (probable)	−0.045 (−0.073; −0.018)	**0.001**	−0.018 (−0.037; 0.002)	0.078	−0.063 (−0.097; −0.029)	**<0.001**	-
Effect of SES on multimorbidity via ACE								
SES	0–1 morbidity	Reference		Reference		Reference		-
	2 + morbidities	−0.029 (−0.057; 0.000)	0.052	−0.009 (−0.014; −0.005)	**<0.001**	−0.038 (−0.067; −0.009)	**0.011**	23.7%*
Effect of SES on multimorbidity via depression								
SES	0–1 morbidity	Reference		Reference		Reference		-
	2 + morbidities	−0.029 (−0.057; 0.000)	0.052	−0.029 (−0.050; −0.007)	**0.009**	−0.053 (−0.088; −0.018)	**0.003**	54.7%*
Effect of SES on multimorbidity via anxiety								
SES	0–1 morbidity	Reference		Reference		Reference		-
	2 + morbidities	−0.029 (−0.057; 0.000)	0.052	0.004 (−0.010; 0.018)	0.58	−0.025 (−0.057; 0.007)	0.13	-
Effect of SES on multimorbidity HL								
SES	0–1 morbidity	Reference		Reference		Reference		-
	2 + morbidities	−0.029 (−0.057; 0.000)	0.052	0.001 (−0.001; 0.004)	0.26	−0.027 (−0.056; 0.002)	0.064	-
Effect of HL on depression via multimorbidity								
HL	PHQ9 < 10	Reference		Reference		Reference		-
	PHQ9 > 10 (probable)	−0.004 (−0.007; −0.001)	**0.024**	0.001 (−0.001; 0.004)	0.26	−0.002 (−0.007; 0.002)	0.25	-
Effect of HL on multimorbidity via depression								
HL	0–1 morbidity	Reference		Reference		Reference		-
	2 + morbidities	0.002 (−0.002; 0.006)	0.24	−0.002 (−0.005; 0.000)	0.052	0.000 (−0.004; 0.005)	0.87	-
Effect of ACE on depression via multimorbidity								
ACE	PHQ9 < 10	Reference		Reference		Reference		-
	PHQ9 > 10 (probable)	0.039 (−0.002; 0.080)	0.065	0.119 (0.059; 0.178)	**<0.001**	0.158 (0.092; 0.223)	**<0.001**	75.3%*
Effect of ACE on multimorbidity via depression								
ACE	0–1 morbidity	Reference		Reference		Reference		-
	2 + morbidities	0.192 (0.155; 0.229)	**<0.001**	0.034 (0.005; 0.064)	**0.021**	0.222 (0.175; 0.269)	**<0.001**	15.3%†
Effect of ACE on depression via anxiety								
ACE	PHQ9 < 10	Reference		Reference		Reference		-
	PHQ9 > 10 (probable)	0.039 (−0.002; 0.080)	0.065	0.343 (0.218; 0.468)	**<0.001**	0.382 (0.251; 0.513)	**<0.001**	89.8%*
Effect of HL on depression via anxiety								
ACE	PHQ9 < 10	Reference		Reference		Reference		-
	PHQ9 > 10 (probable)	−0.004 (−0.007; −0.001)	**0.024**	−0.003 (−0.015; 0.008)	0.58	−0.007 (−0.199; 0.005)	0.24	-

*n*, number of participants; SES, socioeconomic status; HL, health literacy. * Full mediation, *p* < 0.05; †partial mediation, *p* < 0.05; ‡ inconsistent mediation, *p* < 0.05. Bold values denote statistical significance.

## Discussion

The results from this nationally representative study indicate that a significant proportion of South African adults are experiencing mental health challenges (post-COVID-19), with over 21% showing signs of probable depression. While the majority (56.0%) fall into the minimal risk category, the presence of nearly one-quarter experiencing mild to severe depression raises concerns about mental health in the population. The overall prevalence of probable depression has, however, decreased compared to previous years (Panel 1 (12) and Panel 2 (13)), suggesting potential improvements in mental health awareness or access to support across the country. However, with the current results showing one in every five South Africans reporting depressive symptoms, there is a substantial need for mental health resources to be upscaled.

The findings reveal significant regional disparities in mental health, with the Eastern Cape reporting the highest prevalence of probable depression at 30.1% – in line with previous findings (26) – while the Free State shows the highest anxiety rates at 29.2%. These variations suggest the need for targeted interventions based on specific regional challenges. The elevated depressive symptoms in the Eastern Cape are likely linked to socioeconomic issues such as high unemployment and poverty (27, 28), alongside substantial exposure to childhood adversity (12). Limited access to mental health services and cultural stigma further exacerbates untreated conditions (17, 27). In contrast, the Free State’s anxiety levels may stem from economic pressures related to agriculture activities and the rapid increase in urbanization (29). Additionally, a high level of childhood adversity in Limpopo indicates a strong connection to current mental health issues (2–6), as confirmed in those reporting such adversities being more likely to experience depression and anxiety. This finding in both univariate and multivariate analyses, was confirmed in Panel 1 (12) Panel 2 (13) and the current panel (Panel 3). The prevalence of household dysfunction as a common type of adversity underscores the necessity for family-centered interventions. Overall, these results emphasize the importance of tailored mental health strategies to address both psychological needs and the social determinants affecting various communities.

The demographic analysis of the respondents indicated that the majority were young adults aged 25–34 years, accounting for 29.6% of the sample. A significant proportion of respondents identified as single (62.9%) and employed (47.2%), with over half (53.1%) having completed high school or an equivalent level of education. These demographic characteristics are consistent with those observed in previous panels, 1 (2021) (12) and 2 (2022) (13), indicating a stable respondent profile over time. This alignment suggests that the findings from the current study can be contextualized within a familiar demographic framework.

Additionally, when assessing the relationship between ACE exposure and the number of comorbidities, the results indicated that respondents reporting 0–1 morbidity were 80% less likely to have experienced ACEs, highlighting a protective effect for those with fewer adverse experiences. Conversely, the likelihood of having two or more morbidities increased significantly with higher ACE scores, confirming previous findings (13) and thus suggesting a compounded risk for individuals facing multiple health challenges. Adverse experiences are linked to an increased risk of adverse health outcomes, as individuals may adopt unhealthy coping strategies (i.e., poor diet and substance abuse) (13, 30, 31). The socioeconomic consequences of childhood adversity (i.e., lower educational attainment and limited job opportunities) may also perpetuate cycles of poverty and stress, further hindering access to healthcare and healthy lifestyle choices (7, 8). These findings therefore emphasize the critical need for targeted interventions aimed at addressing the long-term consequences of childhood adversity, as they not only affect mental health but also correlate with higher rates of multimorbidity in the population.

We further assessed the complex interplay between health literacy, multimorbidity, and mental health outcomes. The results indicate that SES significantly affects depression, while childhood adversity directly influences multimorbidity. Individuals with lower SES face increased stressors such as financial instability and limited access to healthcare, leading to feelings of hopelessness and social isolation that may elevate depression risk (7, 8). Similarly, childhood trauma, neglect, and/or dysfunction is known to increase the likelihood of developing multimorbidity in later life (13). It is speculated that stress from these adversities, along with unhealthy coping strategies (i.e., poor diet and physical inactivity), may further heighten the risk of multimorbidity (15). Our results further suggest that health literacy partially mediates the relationship between SES and mental health (i.e., depression). Individuals with lower SES often lack access to education and healthcare, making it difficult to comprehend health information and navigate the healthcare system (1). Moreover, low health literacy could potentially prevent individuals from recognizing depressive symptoms and understanding the urgent need for assistance (32), thus prolonging suffering. In contrast, improving health literacy may empower individuals to make informed health decisions and access the suitable resources (14), breaking the cycle of low SES and depression. Our complex results also suggest that childhood adversity and depression in adulthood fully mediates the relationship between SES and multimorbidity in South African adults. Childhood adversity is known to restrict educational and economic opportunities in those most affected, perpetuating low SES and compounding health challenges (7, 8), which underscores the critical role that past adverse experiences play in shaping both mental and physical health trajectories. Specifically, we acknowledge that although the proportion of variance explained in some pathways (i.e., the relationship between SES and depression via health literacy or ACEs) is modest, these mediations may still hold public health relevance – especially in resource-constrained settings where even small gains in health literacy can have meaningful effects. Taken together, these findings underscore the pressing need for integrated interventions that simultaneously target mental and physical health, particularly among socioeconomically disadvantaged populations.

This repeated cross-sectional study should be interpreted considering its strengths and limitations. A key strength of the study is its use of nationally representative data, encompassing respondents from all nine provinces of South Africa, with weights applied to ensure the sample reflects the adult population of the country, however, any stratified analyses represented, is reflective of relative estimates based on the study sample, but not necessarily precise population estimates. Data collection was further strengthened by the extensive training provided to the field staff. However, a potential limitation is that COVID-related information was not included in the survey and given that COVID-19 is known to affect various aspects of health, this absence should be acknowledged as a limitation of the study. Additionally, this national survey includes the use of self-reported questionnaires which may pose recall bias (33). Additionally, the exclusion of individuals with possible cognitive impairment – due to informed consent procedures – may have led to selection bias, particularly as cognitive function is closely linked to health literacy. This may limit the generalisability of our findings to the broader population. Lastly, the HLS-EU-Q47 tool used to measure health literacy has not yet been validated in the South African context. Lastly, the use of gSEM in this cross-sectional study limits causal inference and temporal ordering, and the absence of longitudinal data restricts insight into dynamic or reciprocal processes. By including measures of mental health, multimorbidity and health literacy, the study illustrates the intricate interplay between mental health, multimorbidity, and the ability to effectively engage with healthcare, over and above contemporary sociodemographics.

### Study implications

This study highlights the complex interplay between SES, childhood adversity, health literacy, and mental and physical health outcomes. The findings suggest that improving health literacy and addressing childhood adversity may help mitigate the mental health and multimorbidity burdens associated with socioeconomic disadvantage. The mediating roles of depression, anxiety, ACE and multimorbidity underscores the need for integrated care models that address both mental and physical health. While the cross-sectional design limits causal inference, the results support targeted, upstream interventions and policies aimed at the social determinants of health (i.e., SES, health literacy) to improve long-term health outcomes.

To conclude, the study reveals that one in five South Africans experience depressive symptoms, with notable differences in the prevalence of mental health across the nine provinces. The study further highlights childhood adversity, particularly household dysfunction, as a significant factor linked to mental health issues and increased multimorbidity. Additionally, health literacy was found to mediate the relationship between SES and depression, suggesting that lower health literacy may heighten vulnerability to mental health challenges. These findings underscore the urgent need for targeted interventions to address childhood adversity, improve health literacy, and enhance mental health resources across regions to reduce the burden of mental health in South Africa.

## Data Availability

The original contributions presented in the study are included in the article/Supplementary material, further inquiries can be directed to the corresponding author.
