# Supplementary material for: Health literacy, multimorbidity and its effect on mental health in South African adults: a repeated cross-sectional nationally representative panel study
Source: Front Public Health. 2025 Aug 20;13:1622005. doi: 10.3389/fpubh.2025.1622005 (PMC12404963; doi:10.3389/fpubh.2025.1622005)
Supplement: Supplementary file 1 [file Table_1.docx]

**Supplementary Table S1. Mental health risk, multimorbidity and health literacy of the South African adult population stratified by province.**

|  | | | **Provinces** | | | | | | | | |
| --- | --- | --- | --- | --- | --- | --- | --- | --- | --- | --- | --- |
|  | | | **WC** | **EC** | **NC** | **FS** | **KZN** | **NW** | **GP** | **MP** | **LP** |
| **Depression (PHQ9)** | **Probable depression** | % | 18.5 | 30.1 | 8.1 | 42.8 | 9.7 | 28.7 | 24.1 | 22.3 | 17.3 |
|  | Minimal | % | 61.9 | 51.4 | 57.5 | 20.6 | 69.9 | 46.0 | 53.0 | 48.5 | 67.8 |
|  | Mild | % | 19.6 | 18.5 | 34.5 | 36.6 | 20.5 | 25.3 | 22.9 | 29.3 | 14.9 |
|  | Moderate | % | 10.5 | 17.9 | 8.1 | 30.0 | 5.9 | 21.0 | 15.6 | 17.8 | 11.7 |
|  | Moderately severe | % | 5.7 | 11.5 | 0.0 | 10.7 | 2.2 | 7.7 | 5.2 | 3.7 | 4.1 |
|  | Severe | % | 2.3 | 0.7 | 0.0 | 2.1 | 1.5 | 0.0 | 3.3 | 0.8 | 1.6 |
| **Anxiety (GAD7)** | **Probable anxiety** | **%** | 15.2 | 19.7 | 3.8 | 29.2 | 5.1 | 12.6 | 17.7 | 7.9 | 9.3 |
|  | Minimal | % | 68.2 | 59.5 | 76.6 | 23.1 | 75.0 | 58.3 | 58.5 | 53.3 | 69.4 |
|  | Mild | % | 16.7 | 20.8 | 19.6 | 47.6 | 19.9 | 29.1 | 23.9 | 38.8 | 21.3 |
|  | Moderate | % | 10.6 | 17.3 | 3.8 | 25.7 | 3.6 | 10.8 | 14.6 | 6.7 | 7.7 |
|  | Severe | % | 4.6 | 2.4 | 0.0 | 3.5 | 1.5 | 1.8 | 3.5 | 1.2 | 1.6 |
| **ACEs** | ACE score | Mean ± SD | 1.49±2.33 | 1.72±2.22 | 1.66±2.12 | 0.81±1.78 | 1.17±2.14 | 1.29±2.25 | 1.76±2.58 | 2.00±2.55 | 2.29±2.26 |
|  | No exposure | % | 55.2 | 42.5 | 40.8 | 74.9 | 62.9 | 62.2 | 49.1 | 41.9 | 29.8 |
|  | Intermediate exposure | % | 26.9 | 39.9 | 44.3 | 15.5 | 26.1 | 25.0 | 32.6 | 36.0 | 43.7 |
|  | High exposure | % | 17.9 | 17.6 | 14.9 | 9.7 | 11.0 | 12.8 | 18.3 | 22.1 | 26.5 |
| **Multimorbidity (chronic conditions incl. mental health)** | Multimorbidity score | Mean ± SD | 0.67±1.28 | 0.83±1.22 | 0.32±0.90 | 0.66±1.2 | 0.46±1.1 | 0.88±1.23 | 0.66±1.26 | 0.64±1.25 | 0.40±0.96 |
|  | 0-1 morbidity | % | 82.1 | 72.4 | 91.7 | 84.4 | 90.2 | 72.9 | 84.2 | 83.5 | 86.2 |
|  | 2 morbidities | % | 9.0 | 11.1 | 3.7 | 7.6 | 4.8 | 19.6 | 7.4 | 10.3 | 6.9 |
|  | 3+ morbidities | % | 9.0 | 11.5 | 4.5 | 8.1 | 5.1 | 7.4 | 8.4 | 6.2 | 6.9 |
| **Health literacy** | Inadequate | % | 4.4 | 16.5 | 4.7 | 6.2 | 10.6 | 5.3 | 9.4 | 0.0 | 7.1 |
|  | Problematic | % | 18.9 | 29.2 | 18.8 | 29.3 | 18.5 | 19.4 | 23.6 | 16.7 | 20.9 |
|  | Sufficient | % | 53.2 | 43.5 | 73.0 | 49.0 | 49.0 | 50.4 | 50.7 | 71.5 | 63.1 |
|  | Excellent | % | 23.5 | 10.8 | 3.5 | 15.6 | 21.9 | 25.0 | 16.3 | 11.8 | 9.0 |

Probable depression was categorised into 5 groups based on scoring in the range 0-4 (minimal), 5-6 (mild), 10-14 (moderate), 15-19 (moderately severe), and 20-27 (severe). Probable anxiety was categorised into four groups based on scoring in the range 0-4 (minimal), 5-9 (mild), 10-14 (moderate), and 15-21 (severe). The ACE score was categorised into 3 exposure groups based on scoring 0 (no exposure); 1-3 (intermediate exposure) and 4-12 (high exposure). Multimorbidity score was categorised into 3 groups based on those respondents who reported null or one ailment (0-1 morbidity); those with comorbidity (2 morbidities); and those with multimorbidity (3+ morbidities). Health literacy was categorised into 4 groups based on scoring in the range 0-25 (inadequate), 26-33 (problematic), 34-42 (sufficient) and 42-50 (excellent). Abbreviations: %: percentage; ACEs: adverse childhood experiences; PHQ9: Patient Health Questionnaire; GAD7: Generalised Anxiety Disorder; WC: Western Province; EC: Eastern Cape; NC: Northern Cape; FS: Free State; KZN: Kwa-Zulu Natal; NW: North West Province; GP: Gauteng Province; MP: Mpumalanga; LP: Limpopo Province.

**Supplementary Table S2. Socio-demographics of the South African adult population stratified by mental and overall health risk.**

|  |  | **Generalised Anxiety Disorder (GAD7)** | | | | | **Patient Health Questionnaire (PHQ9)** | | | | | | **ACEs** | | | **Multimorbidity**  **(chronic conditions incl. mental health)** | | | **Health literacy** | | | |
| --- | --- | --- | --- | --- | --- | --- | --- | --- | --- | --- | --- | --- | --- | --- | --- | --- | --- | --- | --- | --- | --- | --- |
|  |  | **Probable anxiety** | **Min** | **Mild** | **Moderate** | **Severe** | **Probable depression** | **Min** | **Mild** | **Moderate** | **Moderately severe** | **Severe** | **No exposure** | **Intermediate exposure** | **High exposure** | **0-1** | **2** | **3+** | **inadequate** | **Problematic** | **Sufficient** | **Excellent** |
| **Age** | | | | | | | | | | | | | | | | | | | | | | |
| 18-24 years | % | 9.6 | 69.3 | 21.1 | 6.2 | 3.4 | 16.9 | 59.9 | 23.2 | 11.5 | 2.9 | 2.4 | 45.9 | 36.8 | 17.3 | 93.2 | 3.3 | 3.5 | 9.8 | 20.0 | 54.1 | 16.1 |
| 25-34 years | % | 12.2 | 62.9 | 24.9 | 9.6 | 2.6 | 21.0 | 57.7 | 21.4 | 14.6 | 4.4 | 2.0 | 49.6 | 31.7 | 18.7 | 92.0 | 4.3 | 3.7 | 6.7 | 21.3 | 56.4 | 15.7 |
| 35-44 years | % | 16.9 | 61.1 | 22.1 | 14.2 | 2.7 | 23.5 | 53.5 | 23.0 | 13.5 | 7.5 | 2.4 | 50.4 | 32.6 | 17.0 | 88.0 | 6.2 | 5.7 | 7.9 | 21.4 | 52.3 | 18.4 |
| 45-54 years | % | 15.8 | 53.6 | 30.6 | 13.0 | 2.8 | 25.4 | 47.2 | 27.5 | 16.0 | 7.8 | 1.6 | 54.5 | 29.3 | 16.2 | 80.0 | 11.7 | 8.3 | 8.6 | 27.8 | 47.8 | 15.8 |
| 55-64 years | % | 13.2 | 62.3 | 24.5 | 12.1 | 1.1 | 19.7 | 60.6 | 19.7 | 15.2 | 4.2 | 0.3 | 57.1 | 28.4 | 14.5 | 69.8 | 14.7 | 15.5 | 8.2 | 23.3 | 49.8 | 18.8 |
| 65+ years | % | 15.3 | 62.0 | 22.7 | 13.2 | 2.1 | 23.8 | 58.8 | 17.4 | 15.8 | 6.9 | 1.1 | 53.2 | 30.4 | 16.3 | 53.1 | 23.5 | 23.4 | 13.1 | 16.3 | 56.0 | 14.6 |
| **Sex** | | | | | | | | | | | | | | | | | | | | | | |
| Male | % | 13.5 | 64.4 | 22.1 | 11.1 | 2.4 | 21.0 | 58.0 | 21.1 | 14.0 | 5.2 | 1.8 | 51.8 | 30.3 | 17.9 | 86.5 | 6.7 | 6.8 | 8.9 | 20.3 | 53.1 | 17.7 |
| Female | % | 14.0 | 59.7 | 26.3 | 11.2 | 2.7 | 22.2 | 54.1 | 23.6 | 14.5 | 5.8 | 1.9 | 50.5 | 33.3 | 16.3 | 81.4 | 10.0 | 8.6 | 7.9 | 23.4 | 53.0 | 15.7 |
| **Marital status** | | | | | | | | | | | | | | | | | | | | | | |
| Single | % | 13.3 | 63.6 | 23.1 | 10.4 | 2.9 | 21.1 | 55.0 | 23.9 | 13.6 | 5.5 | 2.0 | 49.3 | 32.8 | 17.9 | 88.6 | 6.1 | 5.3 | 8.4 | 20.5 | 55.2 | 15.9 |
| Married/Co-habit | % | 13.3 | 62.7 | 24.0 | 11.2 | 2.1 | 22.0 | 59.1 | 19.0 | 14.7 | 5.5 | 1.8 | 54.3 | 31.0 | 14.8 | 81.6 | 9.2 | 9.2 | 6.3 | 23.5 | 50.8 | 19.4 |
| Widowed/Divorced/Separated | % | 17.7 | 49.5 | 32.8 | 15.7 | 2.0 | 23.9 | 50.7 | 25.4 | 16.7 | 6.5 | 0.7 | 51.0 | 28.9 | 20.0 | 62.6 | 19.8 | 17.7 | 15.5 | 25.3 | 47.6 | 11.6 |
| **Employment** | | | | | | | | | | | | | | | | | | | | | | |
| Unemployed | % | 12.7 | 57.6 | 26.8 | 12.5 | 3.1 | 25.3 | 49.6 | 25.1 | 15.7 | 7.7 | 1.9 | 47.5 | 35.8 | 16.7 | 85.4 | 7.6 | 7.1 | 7.1 | 22.9 | 52.5 | 17.6 |
| Employed | % | 15.6 | 65.6 | 21.6 | 10.5 | 2.2 | 18.5 | 59.9 | 21.6 | 12.7 | 3.9 | 2.0 | 54.9 | 28.4 | 16.7 | 88.0 | 6.3 | 5.8 | 8.1 | 22.9 | 51.7 | 17.3 |
| Student | % | 10.1 | 69.5 | 20.4 | 7.1 | 2.9 | 16.9 | 67.1 | 16.0 | 11.8 | 3.3 | 1.8 | 49.7 | 33.5 | 16.8 | 91.7 | 4.4 | 3.9 | 14.3 | 17.4 | 51.8 | 16.4 |
| Retired | % | 13.0 | 58.4 | 28.5 | 11.4 | 1.6 | 23.6 | 56.8 | 19.5 | 17.1 | 5.8 | 0.9 | 51.1 | 30.4 | 20.0 | 55.8 | 23.0 | 21.1 | 11.2 | 16.9 | 61.2 | 10.7 |
| **Education** | | | | | | | | | | | | | | | | | | | | | | |
| Uneducated/ Partial primary | % | 12.0 | 46.4 | 41.7 | 11.0 | 1.0 | 23.3 | 47.3 | 29.5 | 16.4 | 5.9 | 1.0 | 51.3 | 38.5 | 10.2 | 48.8 | 29.0 | 22.1 | 12.4 | 22.1 | 58.2 | 7.3 |
| Primary school | % | 18.3 | 58.1 | 23.6 | 14.5 | 3.8 | 22.5 | 52.9 | 24.6 | 14.8 | 7.7 | 0.0 | 30.7 | 44.0 | 25.3 | 62.0 | 21.4 | 16.6 | 16.1 | 31.7 | 34.8 | 17.4 |
| Partial secondary | % | 16.4 | 56.7 | 26.9 | 13.0 | 3.4 | 28.0 | 49.1 | 22.8 | 18.0 | 7.7 | 2.3 | 46.4 | 32.2 | 21.4 | 80.5 | 9.6 | 9.8 | 7.8 | 25.6 | 52.1 | 14.4 |
| NSC/Short course | % | 12.6 | 64.7 | 22.7 | 10.3 | 2.3 | 18.9 | 58.7 | 22.4 | 12.4 | 4.8 | 1.7 | 54.6 | 30.0 | 15.4 | 89.6 | 5.8 | 4.6 | 8.5 | 21.5 | 53.5 | 16.5 |
| Tertiary | % | 12.0 | 67.2 | 20.9 | 10.0 | 1.9 | 18.3 | 62.4 | 19.3 | 12.9 | 3.4 | 2.0 | 53.4 | 32.6 | 14.0 | 84.8 | 6.7 | 8.5 | 6.1 | 14.1 | 56.5 | 23.2 |
| **Urbanicity** | | | | | | | | | | | | | | | | | | | | | | |
| Metropolitan | % | 15.6 | 62.7 | 21.8 | 11.7 | 3.9 | 21.4 | 57.2 | 21.3 | 13.1 | 5.9 | 2.4 | 54.0 | 30.1 | 15.9 | 84.9 | 7.5 | 7.6 | 9.0 | 22.7 | 51.3 | 17.0 |
| City/Town | % | 14.9 | 59.5 | 25.6 | 12.8 | 2.1 | 22.7 | 53.7 | 23.6 | 14.8 | 6.1 | 1.8 | 52.5 | 30.5 | 17.0 | 82.0 | 8.4 | 9.6 | 7.3 | 20.8 | 53.2 | 18.6 |
| Rural/Village | % | 10.2 | 63.2 | 26.6 | 9.0 | 1.2 | 21.0 | 56.3 | 22.8 | 15.4 | 4.6 | 1.1 | 46.0 | 35.4 | 18.6 | 84.1 | 9.7 | 6.3 | 8.5 | 21.9 | 55.2 | 14.3 |
| **Socio-economic status** | | | | | | | | | | | | | | | | | | | | | | |
| Lower tertile | % | 14.7 | 58.8 | 26.5 | 11.9 | 2.8 | 24.9 | 52.1 | 23.1 | 17.3 | 6.2 | 1.5 | 46.2 | 34.5 | 19.2 | 82.3 | 10.1 | 7.6 | 9.1 | 25.7 | 51.9 | 13.4 |
| Middle tertile | % | 13.6 | 62.2 | 24.2 | 11.3 | 2.3 | 21.4 | 55.2 | 23.4 | 13.6 | 6.1 | 1.7 | 51.1 | 32.0 | 16.9 | 84.2 | 8.3 | 7.5 | 9.2 | 19.8 | 55.4 | 15.7 |
| Upper tertile | % | 12.5 | 66.2 | 21.2 | 9.9 | 2.6 | 17.3 | 62.8 | 19.9 | 10.9 | 3.8 | 2.6 | 58.2 | 27.7 | 14.1 | 85.6 | 6.1 | 8.3 | 6.2 | 19.8 | 51.0 | 23.0 |

Probable depression was categorised into 5 groups based on scoring in the range 0-4 (minimal), 5-6 (mild), 10-14 (moderate), 15-19 (moderately severe), and 20-27 (severe). Probable anxiety was categorised into four groups based on scoring in the range 0-4 (minimal), 5-9 (mild), 10-14 (moderate), and 15-21 (severe). The ACE score was categorised into 3 exposure groups based on scoring 0 (no exposure); 1-3 (intermediate exposure) and 4-12 (high exposure). Multimorbidity score was categorised into 3 groups based on those respondents who reported null or one ailment (0-1 morbidity); those with comorbidity (2 morbidities); and those with multimorbidity (3+ morbidities). Health literacy was categorised into 4 groups based on scoring in the range 0-25 (inadequate), 26-33 (problematic), 34-42 (sufficient) and 42-50 (excellent). Abbreviations: %: percentage; ACEs: adverse childhood experiences; PHQ9: Patient Health Questionnaire; GAD7: Generalised Anxiety Disorder.

**Supplementary Table S3: Logistic regressions to determine the odds of having probable depression or probable anxiety in adulthood.**

|  |  |  | **Depression binary (PHQ-9)**  (*n*= 3171) | | | **Anxiety binary (GAD-7)**  (*n*= 3171) | | |
| --- | --- | --- | --- | --- | --- | --- | --- | --- |
|  |  |  | **OR** | **(95 % Cl)** | ***p* value** | **OR** | **(95 % Cl)** | ***p* value** |
| **Model 1** | ACE | Score | 1.126 | (1.126; 1.126) | **<0.001** | 1.122 | (1.122; 1.122) | **<0.001** |
| **Model 2** | ACE | Score | 1.123 | (1.123; 1.124) | **<0.001** | 1.122 | (1.121; 1.122) | **<0.001** |
|  | Age | Years | 1.008 | (1.008; 1.008) | **<0.001** | 1.015 | (1.015; 1.015) | **<0.001** |
|  | Sex | Male | (reference) | | | (reference) | | |
|  |  | Female | 1.001 | (1.000; 1.003) | **<0.001** | 0.985 | (0.984; 0.987) | **<0.001** |
|  | Education | Uneducated/Partial primary | (reference) | | | (reference) | | |
|  |  | Primary | 0.816 | (0.811; 0.820) | **<0.001** | 1.360 | (1.351; 1.369) | **<0.001** |
|  |  | Partial secondary | 1.340 | (1.334; 1.345) | **<0.001** | 1.414 | (1.407; 1.422) | **<0.001** |
|  |  | NSC/Short course | 0.947 | (0.943; 0.952) | **<0.001** | 1.193 | (1.186; 1.199) | **<0.001** |
|  |  | Tertiary | 0.999 | (0.994; 1.004) | **<0.001** | 1.184 | (1.177; 1.191) | **<0.001** |
|  | SES | Score | 0.959 | (0.959; 0.959) | **<0.001** | 0.961 | (0.961; 0.961) | **<0.001** |
|  | Employment | Unemployed | (reference) | | | (reference) | | |
|  |  | Employed | 0.715 | (0.714; 0.716) | **<0.001** | 0.796 | (0.794; 0.798) | **<0.001** |
|  |  | Student | 0.754 | (0.751; 0.757) | **<0.001** | 0.826 | (0.822; 0.830) | **<0.001** |
|  |  | Retired | 0.694 | (0.692; 0.696) | **<0.001** | 0.478 | (0.476; 0.480) | **<0.001** |
|  | Marital status | Single | (reference) | | | (reference) | | |
|  |  | Married/Co-habit | 1.076 | (1.074; 1.078) | **<0.001** | 0.924 | (0.922; 0.926) | **<0.001** |
|  |  | Widowed/Divorced/Separated | 1.017 | (1.014; 1.020) | **<0.001** | 1.259 | (1.254; 1.263) | **<0.001** |
|  | Urbanicity | Metropolitan | (reference) | | | (reference) | | |
|  |  | City/Towns | 0.944 | (0.942; 0.946) | **<0.001** | 0.843 | (0.841; 0.844) | **<0.001** |
|  |  | Rural/Village | 0.733 | (0.732; 0.735) | **<0.001** | 0.472 | (0.471; 0.473) | **<0.001** |

Model 1: regression unadjusted. Model 2: regression adjusted for socio-demographics. Abbreviations: *n* – number of participants; OR: odds ratio; ACE: adverse childhood experiences; SES: socioeconomic status; PHQ-9: Patient Health Questionnaire; GAD-7: Generalized Anxiety Disorder. Bold values denote statistical significance (p<0.05).

**Supplementary Table S4: Logistic regressions to determine the odds of multimorbidity (chronic conditions excl. mental health) in adulthood.**

|  |  |  | **Multimorbidity (chronic conditions excl. mental health)**  **0-1 morbidities**  (*n*=3018) | | | **Multimorbidity (chronic conditions excl. mental health)**  **2 morbidities**  (*n*=221) | | | **Multimorbidity (chronic conditions excl. mental health)**  **3+ morbidities**  (*n*=201) | | |
| --- | --- | --- | --- | --- | --- | --- | --- | --- | --- | --- | --- |
|  |  |  | **OR** | **(95 % Cl)** | ***p* value** | **OR** | **(95 % Cl)** | ***p* value** | **OR** | **(95 % Cl)** | ***p* value** |
| **Model 1** | ACE | Score | 0.832 | (0.832; 0.832) | **<0.001** | 1.173 | (1.173; 1.174) | **<0.001** | 1.172 | (1.172; 1.172) | **<0.001** |
| **Model 2** | ACE | Score | 0.796 | (0.796; 0.796) | **<0.001** | 1.193 | (1.193; 1.194) | **<0.001** | 1.205 | (1.205; 1.206) | **<0.001** |
|  | Age | Years | 0.944 | (0.944; 0.944) | **<0.001** | 1.047 | (1.047; 1.047) | **<0.001** | 1.052 | (1.052; 1.053) | **<0.001** |
|  | Sex | Male | | (reference) | | (reference) | | |  | (reference) | |
|  |  | Female | 0.713 | (0.712; 0.715) | **<0.001** | 1.430 | (1.427; 1.434) | **<0.001** | 1.217 | (1.214; 1.220) | **<0.001** |
|  | Education | Uneducated/Partial primary | | (reference) | | (reference) | | |  | (reference) | |
|  |  | Primary | 1.254 | (1.247; 1.260) | **<0.001** | 0.847 | (0.842; 0.852) | **<0.001** | 0.774 | (0.769; 0.779) | **<0.001** |
|  |  | Partial secondary | 1.806 | (1.799; 1.813) | **<0.001** | 0.581 | (0.578; 0.583) | **<0.001** | 0.763 | (0.759; 0.766) | **<0.001** |
|  |  | NSC/Short course | 2.415 | (2.405; 2.426) | **<0.001** | 0.569 | (0.566; 0.572) | **<0.001** | 0.445 | (0.442; 0.447) | **<0.001** |
|  |  | Tertiary | 1.569 | (1.562; 1.577) | **<0.001** | 0.577 | (0.573; 0.580) | **<0.001** | 0.921 | (0.915; 0.926) | **<0.001** |
|  | SES | Score | 0.992 | (0.992; 0.992) | **<0.001** | 0.973 | (0.973; 0.973) | **<0.001** | 1.037 | (1.036; 1.037) | **<0.001** |
|  | Employment | Unemployed | | (reference) | | (reference) | | |  | (reference) | |
|  |  | Employed | 1.338 | (1.335; 1.341) | **<0.001** | 0.902 | (0.900; 0.905) | **<0.001** | 0.677 | (0.675; 0.679) | **<0.001** |
|  |  | Student | 0.616 | (0.612; 0.619) | **<0.001** | 1.438 | (1.427; 1.448) | **<0.001** | 1.497 | (1.486; 1.509) | **<0.001** |
|  |  | Retired | 1.100 | (1.096; 1.104) | **<0.001** | 1.033 | (1.029; 1.037) | **<0.001** | 0.786 | (0.783; 0.790) | **<0.001** |
|  | Marital status | Single | | (reference) | | (reference) | | |  | (reference) | |
|  |  | Married/Co-habit | 0.996 | (0.993; 0.998) | **<0.001** | 1.015 | (1.012; 1.018) | **<0.001** | 1.038 | (1.034; 1.041) | **<0.001** |
|  |  | Widowed/Divorced/Separated | 0.890 | (0.888; 0.893) | **<0.001** | 1.046 | (1.042; 1.050) | **<0.001** | 1.124 | (1.119; 1.128) | **<0.001** |
|  | Urbanicity | Metropolitan | | (reference) | | (reference) | | |  | (reference) | |
|  |  | City/Towns | 0.898 | (0.896; 0.900) | **<0.001** | 1.004 | (1.001; 1.007) | **<0.001** | 1.198 | (1.195; 1.202) | **<0.001** |
|  |  | Rural/Village | 1.287 | (1.283; 1.290) | **<0.001** | 0.867 | (0.865; 0.870) | **<0.001** | 0.741 | (0.738; 0.744) | **<0.001** |

Model 1: regression unadjusted. Model 2: regression adjusted for socio-demographics. Abbreviations: *n* – number of participants; SES: socioeconomic status. Bold values denote statistical significance (p<0.05). Multimorbidity score was categorised into 3 groups based on those respondents who reported null or one ailment (0-1 morbidity); those with comorbidity (2 morbidities); and those with multimorbidity (3+ morbidities).
